# Identification of five B-type response regulators as members of a multistep phosphorelay system interacting with histidine-containing phosphotransfer partners of *Populus* osmosensor

**DOI:** 10.1186/1471-2229-12-241

**Published:** 2012-12-19

**Authors:** Lucie Bertheau, Françoise Chefdor, Grégory Guirimand, Vincent Courdavault, Christiane Depierreux, Domenico Morabito, Franck Brignolas, François Héricourt, Sabine Carpin

**Affiliations:** 1 Université d’Orléans, UFR-Faculté des Sciences, UPRES EA 1207, Laboratoire de Biologie des Ligneux et des Grandes Cultures (LBLGC), BP 6759, Orléans, F-45067, France; 2 INRA, USC1328, Arbres et Réponses aux Contraintes Hydriques et Environnementales (ARCHE), BP 6759, Orléans, F-45067, France; 3 Université François-Rabelais de Tours, EA 2106, Biomolécules et Biotechnologies Végétales, 31 avenue Monge, Tours, 37200, France

**Keywords:** Response Regulator (RR), Histidine-containing Phosphotransfer protein (HPt), Osmosensing pathway, *Populus*

## Abstract

**Background:**

In plants, the multistep phosphorelay signaling pathway mediates responses to environmental factors and plant hormones. This system is composed of three successive partners: hybrid Histidine-aspartate Kinases (HKs), Histidine-containing Phosphotransfer proteins (HPts), and Response Regulators (RRs). Among the third partners, B-type RR family members are the final output elements of the pathway; they act as transcription factors and clearly play a pivotal role in the early response to cytokinin in *Arabidopsis*. While interactions studies between partners belonging to the multistep phosphorelay system are mainly focused on protagonists involved in cytokinin or ethylene pathways, very few reports are available concerning partners of osmotic stress signaling pathway.

**Results:**

In *Populus*, we identified eight B-type RR proteins, RR12-16, 19, 21 and 22 in the Dorskamp genotype. To assess HPt/B-type RR interactions and consequently determine potential third partners in the osmosensing multistep phosphorelay system, we performed global yeast two-hybrid (Y2H) assays in combination with Bimolecular Fluorescence Complementation (BiFC) assays in plant cells. We found that all B-type RRs are able to interact with HPt predominant partners (HPt2, 7 and 9) of HK1, which is putatively involved in the osmosensing pathway. However, different profiles of interaction are observed depending on the studied HPt. HPt/RR interactions displayed a nuclear localization, while the nuclear and cytosolic localization of HPt and nuclear localization of RR proteins were validated. Although the nuclear localization of HPt/RR interaction was expected, this work constitutes the first evidence of such an interaction in plants. Furthermore, the pertinence of this partnership is reinforced by highlighting a co-expression of B-type RR transcripts and the other partners (HK1 and HPts) belonging to a potential osmosensing pathway.

**Conclusion:**

Based on the interaction studies between identified B-type RR and HPt proteins, and the co-expression analysis of transcripts of these potential partners in poplar organs, our results favor the model that RR12, 13, 14, 16 and 19 are able to interact with the main partners of HK1, HPt2, 7 and 9, and this HPt/RR interaction occurs within the nucleus. On the whole, the five B-type RRs of interest could be third protagonists putatively involved in the osmosensing signaling pathway in *Populus*.

## Background

Plants display sophisticated sensing and signaling systems which elicit a variety of responses to environmental signals such as drought or osmotic stress, and to plant hormones including cytokinin. The resulting intracellular signal transduction relies notably on phosphorylation events, which are mediated by multistep phosphorelay signaling. This system involves three components: a hybrid Histidine-aspartate Kinase (HK) receptor, Histidine-containing Phosphotransfer proteins (HPt) and Response Regulators (RR). One of the best characterized corresponding systems is the osmo-responsive pathway operating in yeast. This system is composed of the HK receptor, Sln1p, the HPt, Ypd1p and the RR, Ssk1p [1,2]. The phosphorelay consists in transferring phosphate from His-to-Asp residues between the different partners. Under hyper-osmolarity conditions, Sln1p is inactive and unphosphorylated, leading to the accumulation of unphosphorylated cytoplasmic RR Ssk1p. This active form of Ssk1p is then able to activate the HOG1 MAP kinase pathway that induces genes expression, leading to cell protection by glycerol synthesis. In *Arabidopsis thaliana*, multistep phosphorelay members similar to the one found in yeast are involved in signaling pathways: *Arabidopsis* histidine kinases (AHKs), *Arabidopsis* histidine-phosphotransfer proteins (AHPs) and *Arabidopsis* response regulators (ARR) [3]. The AHK family consists of six hybrid histidine protein kinases, AHK1, AHK2, AHK3, AHK4 (CRE1/WOL), AHK5 and CKI1. These latters, CKI1 and AHK5, are involved in megagametophyte development [4-6] and stomatal closure [7] respectively. This family also includes the cytokinin receptors AHK2, AHK3, AHK4 [8-11] and AHK1 which is the first partner of the osmosensing pathway displaying an osmosensor function in both models, yeast and *Arabidopsis*[12,13]. This latter also plays a role in the regulation of desiccation processes during seed maturation [14]. Contrary to AHK1, cytokinin receptors AHK2, 3 and 4 function as negative regulators of osmotic stress [13]. Concerning the second partner, 5 AHPs mediate the phosphorelay between AHKs and ARRs. Urao *et al.* found that the osmosensor receptor, AHK1, is only able to interact with one HPt protein AHP2 [15]. An interaction network study of multistep phosphorelay signaling pathway members performed by yeast two-hybrid assays showed interactions of AHP2 with some ARRs [16,17]. On the basis of a structural comparaison of amino acid sequences, the members of the ARR family were subdivided into four distinct groups including A-type, B-type, C-type and pseudo-RRs [3,18]. Among these groups, the B-type RR family members are assumed to function as crucial transcriptional regulators in the His-to-Asp phosphorelay signal transduction network. Such RRs are composed of a phosphate receiver domain with the conserved D-D-K motif (RR domain), and a large C-terminal extension mediating sequence specific DNA-binding domain referred to originally as the B motif [3,19]. According to Riechmann’s classification [20], the B motif appears to be a representative of the plant single Myb-related domains, which belong to the GARP subfamily. The GARP domain or B motif is specific to transcription factors found only in plant, and its name derives from its discovery inside the maize GOLDEN2 gene sequence, the B-type ARR proteins from *Arabidopsis*, and Psr1 from *Chlamydomonas*[20]. The C-terminal extension contains a transcriptional activation region which is rich both in proline and glutamine residues, as it is usually met in transcriptional activators [21] and also nuclear localization signals (NLSs) responsible for the B-type RRs targeting to the nucleus [22-27]. Within the eleven members of B-type RR family identified in *Arabidopsis* (ARR1, 2, 10, 11, 12, 13, 14, 18, 19, 20, 21), some interacting partners of AHP2 [16,17,28] have been shown to be associated with cytokinin signal transduction [29-32]. This signal is relayed from membrane to nucleus where these RRs function as transcription factors that operate in the last step of the primary cytokinin response pathway. Although *B-type RR* genes expression is not cytokinin inducible, B-type RRs function as positive regulators of the cytokinin signaling pathway [31-34] by enhancing transcription of cytokinin target genes, including A-type ARRs [25,29], which act in turn as negative regulators allowing a feedback control of the pathway [25,35]. While B-type RRs involvement in cytokinin signaling pathway has been studied in detail, little is known about their role in osmosensing signaling pathway in other plants than *Arabidopsis* and more particularly in woody plants. In *Populus trichocarpa*, 11 A-type, 11 B-type, 10 C-type and 17 pseudo RRs have been identified to date [18,36]. A transcriptomic analysis revealed that some genes corresponding to cytokinin signaling pathway components (HK, HPt and RR) were regulated during shoot organogenesis in hybrid poplar 717-1B4 genotype [37]. Moreover, in the same genotype, Ramirez-Carvajal *et al*. [38], showed that RR13 acted downstream of cytokinin pathway by repressing adventitious root formation. In poplar Dorskamp genotype, we identified a putative osmosensor, HK1 [39], as well as ten HPts (HPt1 to HPt10). A recent study led us to propose HPt2, 7 and 9 as specific partners of HK1 in the *Populus* osmosensing signaling pathway [40].

To characterize in more detail the molecular mechanisms involved in the poplar osmosensing pathway, we undertook to analyze potential interactions of the three HPt partners of HK1 (HPt2, 7 and 9) with the different B-type RRs. As a consequence, we isolated eight B-type RRs in our poplar genotype and performed interaction tests by yeast two-hybrid (Y2H) and Bimolecular Fluorescence Complementation (BiFC) assays. These tests showed that the three HPts interact with eight B-type RRs and presented distinct interaction profiles based on different level of reporter gene activation. The interaction study *in planta* for some B-type RRs by BiFC assays confirmed a nuclear localization of HPt/B-RR interactions. The co-expression of some B-type RR and HPt transcripts in same poplar organs led us to highlight five of them as potential partners for these three HPt proteins.

## Results

### Isolation of eight poplar B-type response regulators

**Figure 1:**
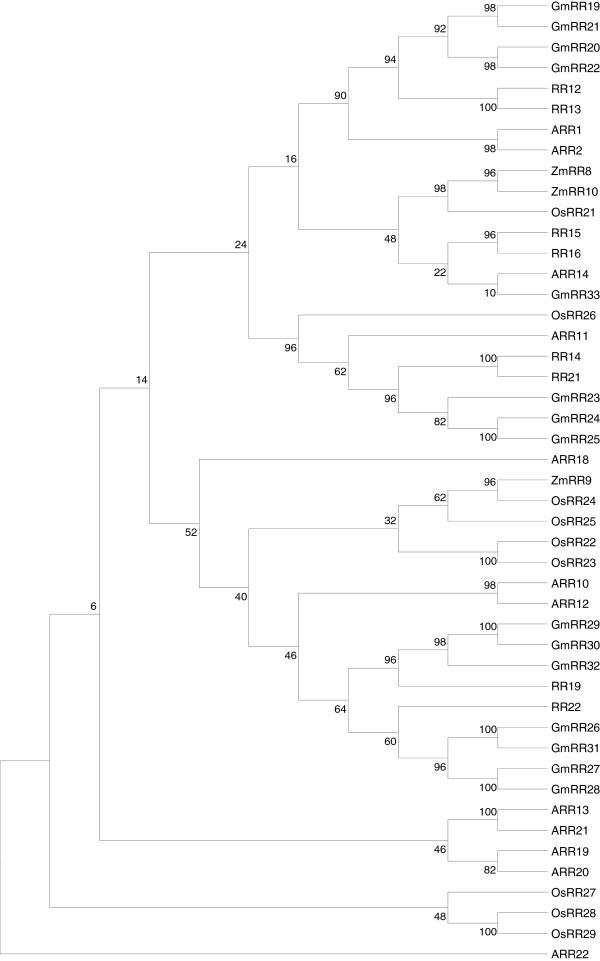
**Phylogenetic tree of B-type RR family members.** The full-length protein sequences of poplar B-type RRs deduced from cDNA sequences (EMBL: FN908138 to FN908145) were aligned using Muscle [41] with those of *Arabidopsis* (ARRs)*,* soybean (GmRRs)*,* maize (ZmRRs) and rice (OsRRs), obtained from respective genome databases (Additional file 2). Maximum likelihood tree reconstructions were performed with MEGA 5 [42] using the JTT substitution model [43]. The C-type ARR22 from *Arabidopsis* was used as outgroup. Numbers indicate bootstrap support (1000 replicates).

On the basis of *Populus* genomic resources, we isolated eight cDNAs encoding B-type RRs from a root cDNA library: RR12, 13, 14, 15, 16, 19, 21, 22 (EMBL: FN908138 to FN908145). We did not succeed in isolating RR17, 18 and 20. Deduced amino acid sequences of these newly isolated poplar B-type RRs share a common structural design composed of the phospho-accepting receiver domain, the GARP DNA-binding domain and two putative NLSs. The phylogenetic relationships of these B-type RRs with those of model plant species, *Arabidopsis thaliana* (ARRs), *Zea mays* (ZmRRs)*, Glycine max* (GmRRs)*,* and *Oryza sativa* (OsRRs), are represented by a rooted tree based on the alignment of B-type RR full-length amino acid sequences (Figure 1). Such analysis revealed that the different B-type RR family members are interspersed in distinct groups independent of species but in most cases, are classified within groups in species-specific pairs.

### HPt2, HPt7 and HPt9 interact with a subset of B-type response regulators in yeast

**Figure 2:**
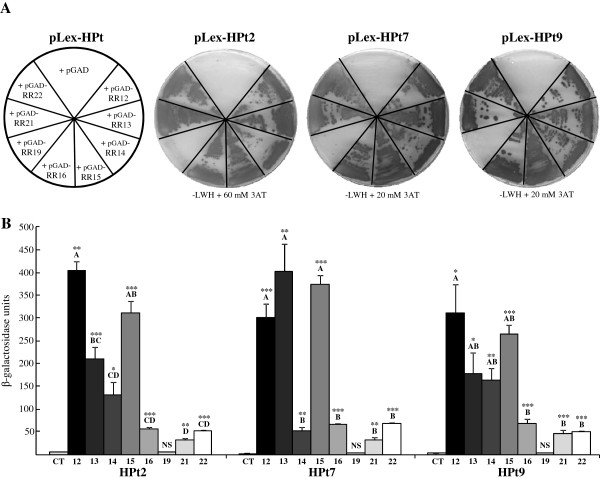
**HPt2, HPt7 and HPt9 interact with a subset of B-type RRs in the yeast two-hybrid system. A**) X-gal assays of HPt/B-type RR interactions. HPts (HPt2, 7, 9) interactions with B-type RRs (RR12, 13, 14, 15, 16, 19, 21 and 22) as indicated and the corresponding X-gal assay on leucine-tryptophan-histidine lacking medium (−LWH) supplemented with 60 (HPt2) or 20 (HPt6, 7, 9) mM of 3AT. **B**) ß-Galactosidase activity measurements of HPt/B-type RR interactions. Means ± standard error of three independent replicates of ß-Galactosidase activity measurements are presented for each HPt/B-type RR interaction. The negative control (CT) of each HPt is the interaction background between HPt2, 7 or 9 and the empty vector pGAD. The mean values of HPt/RR interactions were compared to their respective negative control by a t-test and asterisks indicate significant differences: *P < 0.05, **P < 0.01, ***P < 0.001 and NS for not significant. The mean values of interaction between HPts and RR12, 13, 14, 15, 16, 21, and 22 are compared by ANOVA followed by Scheffe test; different letters indicate significant differences (P ≤ 0.05).

To study interactions of the three HPts of interest (HPt2, 7, 9) with the eight isolated B-type RRs, we conducted a Y2H analysis. Since B-type RR expression in the “bait” configuration led to a strong autoactivation of the first reporter gene (*HIS3*) (data not shown), all the B-type RRs were used as a “prey” while the three HPts tested were cloned in the “bait” vector. This analysis revealed interactions of the three HPts with seven B-type RRs: RR12, RR13, RR14, RR15, RR16, RR21 and RR22. No interaction was detected between HPts and RR19 using this system (Figure 2A). These results were confirmed by the estimation of the ß-Galactosidase activity corresponding to the second reporter gene activation (Figure 2B). As a control of the interaction background, we used each pLex-HPt in combination with the empty pGAD vector that did not result in significant ß-Galactosidase activity. As expected, similar low levels of activity were also obtained when testing the interaction of the three HPts with RR19. By contrast, we observed that the other RRs interacted significantly with the three HPts. Indeed, for HPt2 and 9 similar patterns of interaction were revealed including a strong ß-Galactosidase activity for RR12, and an intermediate one for RR13, 14 and 15 and a weaker one for RR16, 21 and 22. On the other hand, HPt7 presented an interaction pattern separating RRs in two groups, the first one with a strong ß-Galactosidase activity (RR12, 13 and 15) and the second one exhibiting a weaker activity (RR14, 16, 21 and 22).

### HPt/RR interactions occur in the nuclear compartment

**Figure 3:**
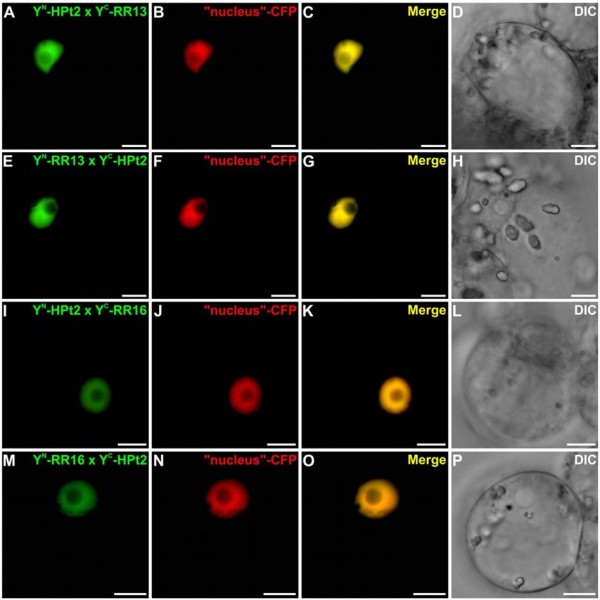
**Analysis of HPt2/RR13 and HPt2/RR16 interactions in *C. roseus *cells using BiFC assays.** Cells were co-transformed using a combination of plasmids expressing HPt2/RR13 (**A-H**) and HPt2/RR16 (**I-P**) as indicated. For each combination, an additional co-transformation was performed with the CFP nuclear marker (**B, F, J, N**). Co-localization of the two fluorescence signals is shown in the merged image (**C, G, K, O**). The morphology was observed by differential interference contrast (**DIC**) microscopy (**D, H, J, P**). Scale bar = 10 μm.

**Figure 4:**
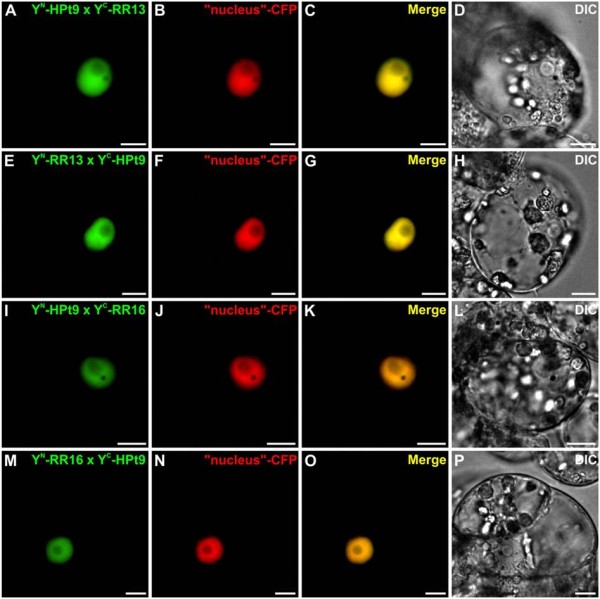
**Analysis of HPt9/RR13 and HPt9/RR16 interactions in *C. roseus *cells using BiFC assays.** Cells were co-transformed using a combination of plasmids expressing HPt9/RR13 (**A-H**) and HPt9/RR16 (**I-P**) as indicated. For each combination, an additional co-transformation was performed with the CFP nuclear marker (**B, F, J, N**). Co-localization of the two fluorescence signals is shown in the merged image (**C, G, K, O**). The morphology was observed by differential interference contrast (**DIC**) microscopy (**D, H, J, P**). Scale bar = 10 μm.

**Figure 5:**
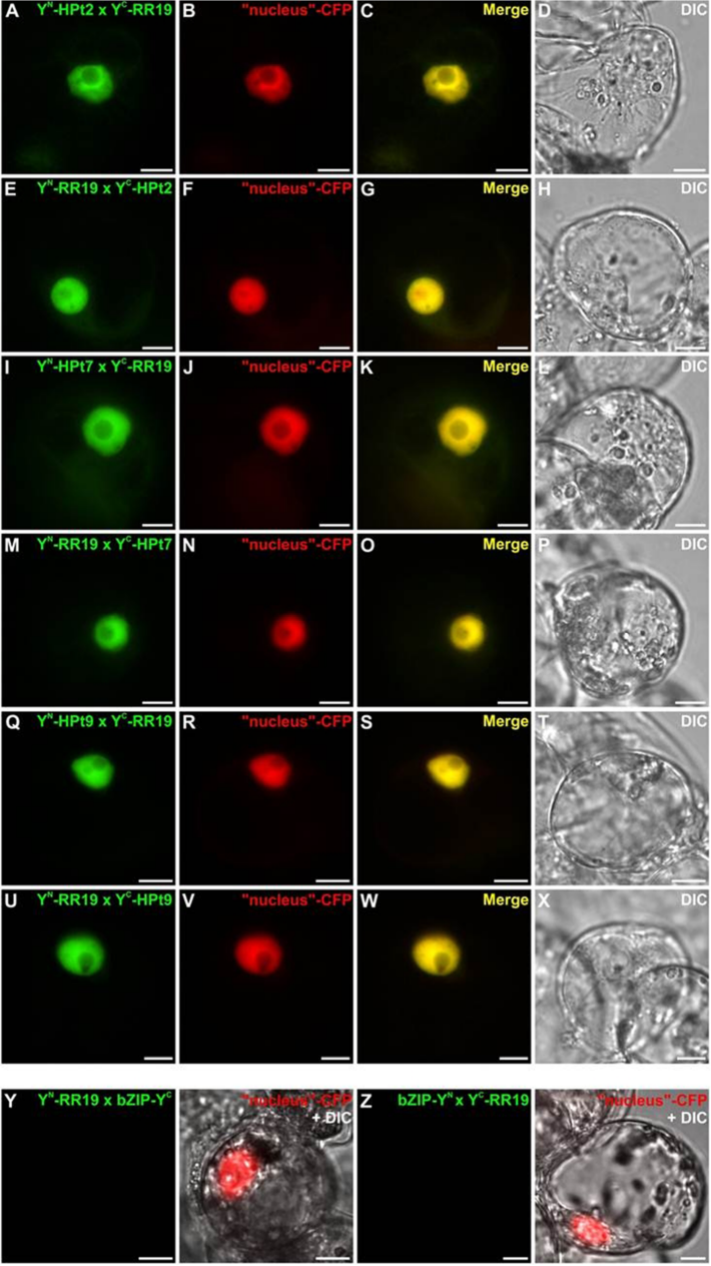
**Analysis of RR19/HPts (HPt2, 7 and 9) interactions in *C. roseus *cells using BiFC assays.** Cells were co-transformed using a combination of plasmids expressing HPt2/RR19 (**A**-**H**), HPt7/RR19 (**I**-**P**) and HPt9/RR19 (**Q**-**X**) as indicated. For each combination, an additional co-transformation was performed with the CFP nuclear marker (**B**, **F**, **J**, **N**, **R**, **V**). Co-localization of the two fluorescence signals is shown in the merged image (**C**, **G**, **K**, **O**, **S**, **W**). The morphology was observed by differential interference contrast (DIC) microscopy (**D**, **H**, **J**, **P**, **T**, **X**). Scale bar = 10 μm. As negative controls, two combinations of plasmids were used: YFP^N^-RR19/bZIP- YFP^C^ and bZIP-YFP^N^/YFP^C^-RR19 (**Ya**-**b**, **Za**-**b**), supplemented by a co-transformation with the CFP nuclear marker. The morphology was observed by differential interference contrast (DIC) microscopy (**Yb**, **Zb**). Scale bar = 10 μm.

**Figure 6:**
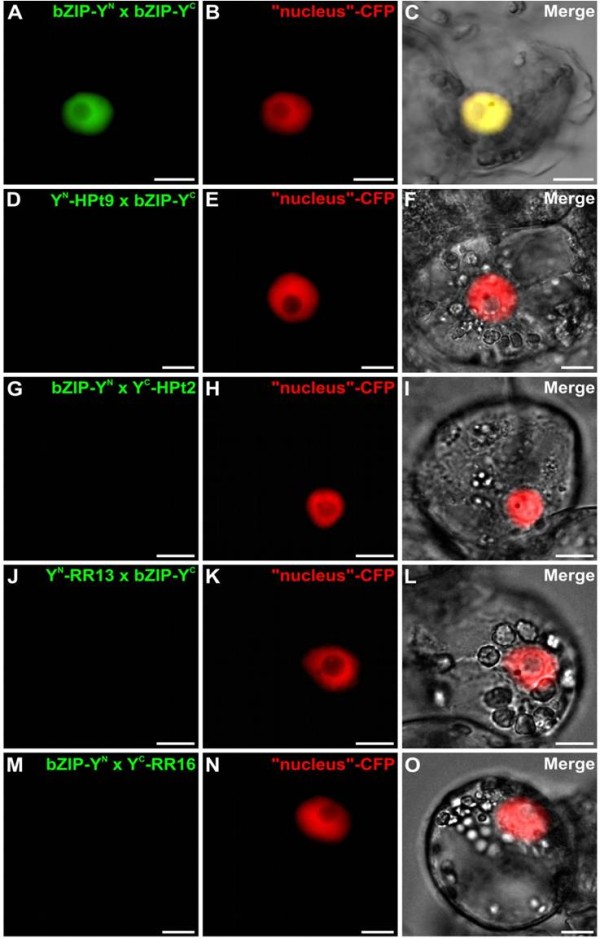
**Controls of BiFC assays in *C. roseus *cells.** Cells were co-transformed using a combination of plasmids as indicated: (**A**-**C**) the split of nuclear transcription factor bZIP as a positive control. An additional co-transformation with the CFP nuclear marker (**B**) confirms the co-localization of the two fluorescence signals (**C**). (**D**-**O**) YFP^N^-HPt9/-RR13 or YFP^C^-HPt2/-RR16 with the split bZIP as a negative control. For each combination, an additional co-transformation with the CFP nuclear marker was performed (**E**, **H**, **K**, **N**). The morphology was observed by differential interference contrast (DIC) microscopy (**C**, **F**, **I**, **L**, **O**). Scale bar = 10 μm.

To confirm and localize *in planta* the HPt/RR interactions observed in Y2H assays, we tested whether HPts can interact with B-type RRs using BiFC assays in *Catharanthus roseus* cells. For such analysis, four detected interactions in the Y2H system were tested: HPt2/RR13, HPt2/RR16, HPt9/RR13 and HPt9/RR16. Furthermore, in order to substantiate the lack of HPt/RR19 interaction in yeast, BiFC assays were conducted to test the interaction between RR19 and HPt2, 7, 9 *in planta*. HPt2, 7, 9, RR13, 16 and 19 coding sequences were fused either to the N-terminal (YFP^N^) or C-terminal (YFP^C^) fragments of yellow fluorescent protein (YFP) at their N-terminal end to produce: YFP^N^-HPts, YFP^C^-HPts and YFP^N^-RRs, YFP^C^-RRs. During co-transformation experiments, the different combinations of HPt2 and RR13 or RR16 constructs led to the formation of a BiFC complex within plant cells (Figure 3A, E, I, M). This signal perfectly merged with the fluorescence of the cyan fluorescent protein (CFP) nucleus marker (Figure 3A-P) indicating the nuclear localization of this interaction. Similar constructs, utilized to test the interactions HPt9/RR13 and HPt9/RR16 using BiFC assays, revealed a YFP reconstitution in both cases (Figure 4A, E, I, M). Both interaction combinations displayed a nuclear fluorescence signal, as demonstrated by the co-localization with the signal of the co-expressed CFP nucleus marker (Figure 4A-P). As regards RR19, the formation of a BiFC complex within plant cells was observed in case of interaction with HPt2 (Figure 5A, E), HPt7 (Figure 5I, M) and HPt9 (Figure 5Q, U). This signal also perfectly merged with the CFP nucleus marker (Figure 5A-X) indicating the nuclear localization of these three interactions. As a positive control, BiFC complex reconstitution was visualized when co-expressing the fusion proteins bZIP-YFP^N^ and bZIP-YFP^C^, confirming a nuclear localization of homodimers of this known transcription factor (Figure 6A-C). By contrast, no YFP reconstitution could be visualized when co-expressing the fusion proteins YFP^C^-HPt2, YFP^N^-HPt9, YFP^N^-RR13, YFP^C^-RR16, YFP^N^ or YFP^C^-RR19 with the fusion protein bZIP-YFP^C^ or bZIP-YFP^N^ (Figure 6D-F, G-I, J-L, M-O and Figure 5Ya-b, Za-b), thereby validating the specificity of interaction between B-type RRs (RR13, 16 and 19) and HPts (HPt2, 7 and 9) *in planta.* This interaction study confirmed *in planta* some of the interactions detected by Y2H analysis, between RR13 or RR16 and HPt2 or HPt9. In contrast to Y2H system, BiFC assays highlighted the ability of RR19 to interact with the three HPts of interest. The totality of these HPt/RR interactions occurs in the nuclear compartment.

**Figure 7:**
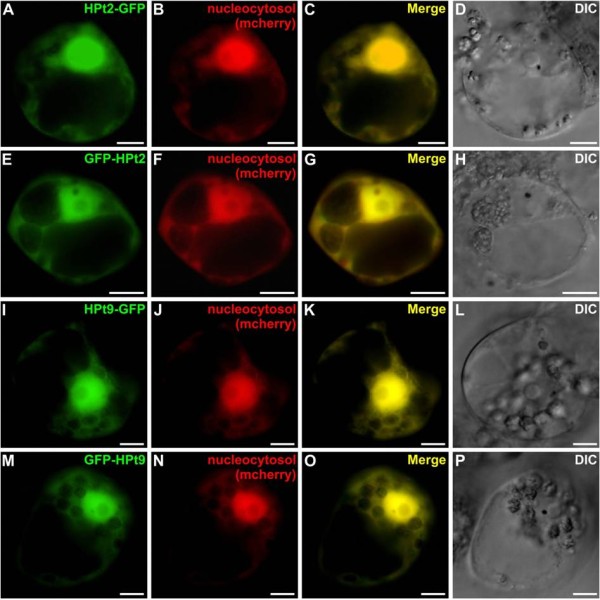
**Nuclear and cytosolic localization of HPt2 and HPt9 in *C. roseus *cells.** Cells were transiently transformed with HPt-GFP (HPt2 (**A**-**D**); HPt9 (**I**-**L**)) or GFP-HPt (HPt2 (**E**-**H**); HPt9 (**M**-**P**)) expressing vectors in combination with the nucleocytosolic-mcherry marker (**B**, **F**, **J**, **N**). Co-localization of the two fluorescence signals is shown in the merged image (**C**, **G**, **K**, **O**). The morphology was observed by differential interference contrast (DIC) microscopy (**D**, **H**, **L**, **P**). Scale bar = 10 μm.

**Figure 8:**
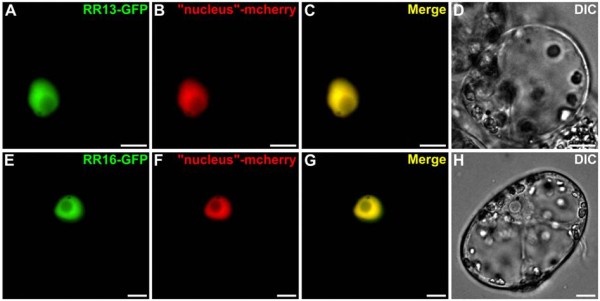
**Nuclear localization of RR13 and RR16 in *C. roseus *cells.** Cells were transiently transformed with RR13-GFP (**A**-**D**) or RR16-GFP (**E**-**H**) expressing vectors in combination with the nuclear-mcherry marker (**B**, **F**). Co-localization of the two fluorescence signals is shown in the merged image (**C**, **G**). The morphology was observed by differential interference contrast (DIC) microscopy (**D**, **H**). Scale bar = 10 μm.

The nuclear localization of these interactions prompted us to examine the subcellular localization of these different partners, HPt2, HPt9, RR13 and RR16, using transient expression of GFP-fusion proteins within *C. roseus* cells. This study showed that HPt2 (Figure 7A-H) and HPt9 (Figure 7I-P) GFP-fusion proteins displayed both a nuclear and cytosolic localization as illustrated by a perfect merge of fluorescence with the mcherry nucleo-cytosolic marker (Figure 7C, G, K, O). By contrast, the RR13 (Figure 8A-D) and RR16 (Figure 8E-H) GFP-fusion proteins were exclusively localized into the nucleus as demonstrated by the co-localization of the GFP fluorescent signal with the signal of the co-expressed mcherry nuclear marker (Figure 8C, G) validating the RR13 and RR16 nuclear localization.

### Expression profile of B-type RRs transcripts in *Populus*

**Figure 9:**
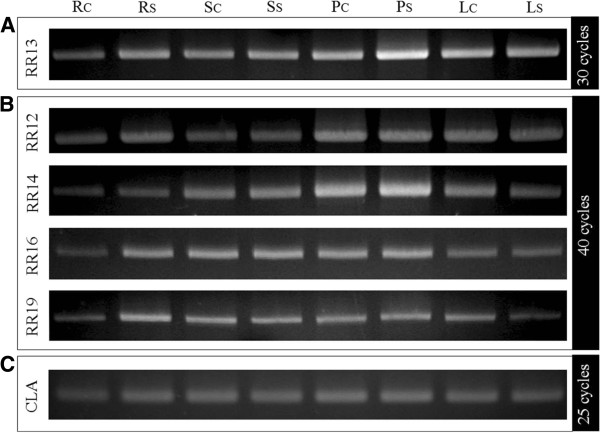
**Expression analysis of RR12, 13, 14, 16, 19 by RT-PCR.** RNAs isolated from roots (R), stems (S), petioles (P) and leaves (L) under control (c) or stressed (s) conditions, were reverse transcribed and used as template for PCR amplification. PCR reactions were performed using *B-type RRs* specific primers under optimal conditions for each primer set (**A**: 30 cycles, **B**: 40 cycles). Expression profile of a housekeeping gene, *Clathrin* (CLA), was realized with 25 cycles of PCR amplification.

The physiological existence of the interactions observed between HPts and B-type RRs relies on the concomitant presence in plant organs of both partners. As a consequence, the distribution of the transcripts of each protein has been evaluated by RT-PCR approaches. For this purpose, RNAs were extracted from various organs (roots, stems, petioles, leaves) of poplar cuttings in control or stressed conditions (treatment by polyethylene glycol 6000 [PEG 6000] at 50 g/L for 10 min duration) and reverse transcribed. The resultant cDNAs were amplified by PCR using specific primers (Additional file 1) for each *B-type RRs* and *Clathrin* gene was used as control (Figure 9C). While the transcripts of *RR13* were detected in all organs and conditions following 30 cycles of amplification (Figure 9A), 10 additional cycles were needed to detect in all organs and conditions the transcripts of *RR12*, *14*, *16* and *19* (Figure 9B). By contrast, the transcripts of *RR15*, *21* and *22* were not detected in these conditions (data not shown). This analysis suggests that among all poplar *B-type RRs*, the transcripts of *RR12*, *13*, *14*, *16* and *19* are more expressed than the others with a more marked expression for *RR13*. Besides, under the conditions of our study, the osmotic constraint applied did not allow to detect marked variations in transcripts expression whatever the *B-type RRs* studied.

## Discussion

The B-type response regulators are the final output elements of the His-to-Asp phosphorelay system. In spite of a systematic identification and characterization of the different partners belonging to these systems in *Arabidopsis*, *Zea mays, Glycine max,* and *Oryza sativa*, only few members of these systems from herbaceous plants have been identified as drought-related actors. In *Arabidopsis,* it has been demonstrated that the hybrid Histidine-aspartate Kinase, AHK1, is a positive regulator of osmotic stress signaling and that *AHK1* gene expression is regulated by salt stress and dehydration [13,14]*.* Moreover, among three authentic AHPs (AHP1, 2 and 3), only AHP2 was shown to be an interacting partner of this osmosensor [15]. However, the B-type RR interacting partners of AHP2 [16,17,28] clearly play a pivotal role in the response to cytokinin [29-32] or to ethylene [44]. Until now, very few information is available concerning an interaction network between osmotic stress signaling partners belonging to the multistep phosphorelay system.

The aim of the present study was to determine, in *Populus*, the potential third partners in osmotic stress signaling by bringing out interactions between B-type RRs and three HPts main interacting partners of the putative osmosensor HK1. Based on a homology approach, we isolated eight cDNAs, from the poplar Dorskamp genotype, encoding RRs sharing the common features of B-type RR family members. A phylogenetic analysis of poplar B-type RRs and several RRs from other model plant species revealed that in most cases, B-type RR family members can be classified independently of species but present pairings within species. In *Arabidopsis*, based on sequence comparisons and expression patterns, the B-type RRs have been divided into three sub-groups: BI, the largest sub-group that contains ARR involved in cytokinin responses [25,29], BII and BIII represented respectively by the pairs ARR13/ARR21 and ARR19/ARR20 [27,33,45]. Our rooted tree produced the same global scheme: the B-type RRs of the sub-group BI of *Arabidopsis* clustered together notably with a high number of B-type RRs from the other species and more particularly with isolated poplar B-type RRs.

We did not succeed in isolating RR17 or 20 which belong to the sub-group II, or the RR18 of sub-group I. Nevertheless, as shown in *P. trichocarpa* genotype, *RR17* and *RR20* are not expressed in roots [36], in agreement with our difficulties in isolating them. On the other hand, in the Dorskamp genotype, we isolated RR14 but not RR18, which were undetected and detected respectively in *P. trichocarpa* genotype. These differential expressions of B-type RRs observed in *P. trichocarpa* and *P. deltoides* (Bartr.) Marsh x *P. nigra* L. Dorskamp genotypes could be explained by their genetic variability.

To identify the partners potentially involved in the osmosensing pathway, we performed a global interaction analysis in yeast between HPts (HPt2, 7, and 9), the main partners of HK1, and the eight isolated B-type RRs. Both qualitative approaches used allowed us to obtain an overview of B-type RR partners of these HPts. Except for RR19, strong interactions between all B-type RRs and the three HPts were detected with different interaction profiles. Based on different activation levels of the second reporter gene (β-galactosidase), three RR groups emerged for HPt2 and HPt9, while the RRs are divided into two groups for HPt7.

To substantiate the partnership observed by Y2H analysis, some HPt/RR interactions were also analyzed *in planta* using BiFC assays*.* Among interactions observed in yeast, two HPts and two B-type RRs were chosen: i) HPt2 and HPt9, that present the strongest interaction with HK1 and seem to be key actors of the osmosensing pathway on the basis of their expression profiles in *Populus*[40], ii) RR13 and RR16, that display strong or weak β-galactosidase activity with the three HPts, respectively. These BiFC assays demonstrated that HPt2 and HPt9 interact with RR13 or RR16 in the *C. roseus* cells. In *Arabidopsis*, an interaction study concerning Cytokinin Response Factors (CRFs) and other members of cytokinin signaling pathway showed that BiFC technique led to a more complete detection of interactions than with the Y2H method [46]. Based on this study and due to the lack of HPt2, 7, 9/RR19 interactions observed in Y2H system, we decided to complete the interaction study by testing the ability of RR19 to interact *in planta* with HPts. As for the CRFs study, the BiFC analysis showed that RR19 displayed interactions with HPt2, 7 and 9. Cutcliff *et al*. [46], speculated that the lack of some interaction in Y2H system can be explained by the absence of plant specific post-translational modifications on protein or plant-specific protein facilitating the interactions of this protein. The whole of HPt/RR interactions *in planta* occurs in the nucleus. To validate this study, we confirmed that HPt2 and 9 have a nuclear and cytosolic localization while the RR13 and 16 are localized in the nucleus. Although the localization of the second and third partners has already been described in *Arabidopsis*[22-27], and that the nuclear localization of HPt/B-type RR interactions is expected, the localization of these interactions was until now only putative. Our study shows clearly this localization for the first time in plant cell.

To reinforce the hypothesis of interactions between the second and third partners *in planta*, an analysis of the concomitant presence of the transcripts of both partners (HPts and B-type RRs) in the same plant organs was performed. A previous analysis of expression of *HK1* and *HPt* genes in four different organs of poplar cuttings under control and osmotic stress conditions revealed that only *HK1*, *HPt2*, *7* and *9* are co-expressed in all organs and conditions tested [40]. In the present study, we also analyzed the B-type RR genes expression in the same poplar cuttings organs and conditions, which led us to show that only *RR12*, *13*, *14*, *16* and *19* are expressed in the same organs and conditions than *HK1* and the three *HPts*. By contrast to HPts, none B-type RR showed an organo-specific expression. However, the absence of *RR15*, *21* and *22* transcripts in organs and conditions tested, even though they are able to interact physically with HPts in yeast, allows us to conclude that these B-type RRs are of lesser importance in the osmosensing signaling pathway in these organs.

The five poplar B-type RRs, RR12, 13, 14, 16 and 19, interacting with HPt2, 7 and 9, the predominant partners of HK1, and concomitantly expressed in the main organs of poplar, could be probably involved in a poplar osmosensing pathway. Nevertheless, some *Arabidopsis* B-type RRs homologous to these five poplar RRs are transcription factors of cytokinin or ethylene signaling pathways, such as ARR2 involved in leaf senescence [47], ethylene response [44] and resistance against pathogens [48]. Consequently, these transcription factors would behave as a set of protagonists linking several signaling pathways to coordinate different developmental processes and stress responses [19]. This hypothesis is consistent with the known cross-talk between cytokinin, ABA and abiotic stresses [13,49,50].

## Conclusion

In the present work, eight B-type RRs have been identified in *Populus* (Dorskamp genotype). In yeast, almost all B-type RRs tested (except RR19) present interactions with the three preferential partners, HPt2, 7, 9, of HK1, a putative poplar osmosensor. Some HPt/RR interactions were confirmed *in planta* by BiFC assays and the ability of RR19 to interact with HPts was only detected *in planta*. All tested HPt/RR interactions displayed a nuclear localization, HPts being nuclear and cytosolic proteins, and B-type RRs nuclear proteins. The co-expression of the downstream signaling partners in poplar organs highlights the relevance of RR12, 13, 14, 16 and 19 as potential third partners involved in the osmosensing pathway in *Populus*. However, their specific implications in transcription of osmotic stress response genes remain to be investigated.

## Methods

### Isolation of B-type RR CDSs and phylogenetic analysis

We used the references of gene from *Populus trichocarpa* B-type RRs (PtRR12 to PtRR22) [36] to search their nucleotidic sequences in JGI *Populus trichocarpa* (v1.1) and designed a specific primers pairs corresponding to each RR (Additional file 1), in order to isolate their CDSs from *Populus deltoides* (Bartr.) Marsh x *P. nigra* L. Dorskamp genotype. PCRs were performed using a root cDNA library constructed with the Marathon cDNA Amplification Kit (Clontech) and Taq Advantage polymerase (Clontech), with primers at a final concentration of 0.2 μM. PCR products were cloned into pGEM-T vector (Promega), sequenced and compared with *P. trichocarpa* B-type RRs sequences using ClustalW [51] as implemented in BIOEDIT version 7.0.9 [52].

Multiple amino acid sequences alignments of all poplar B-type RRs and those of *Arabidopsis thaliana, Zea mays, Glycine max, Oryza sativa* (Additional file 2) were carried out using Muscle [41] incorporated within the software MEGA 5 [42]. Maximum likelihood tree reconstructions were performed with MEGA 5 and generated using the Jones-Taylor-Thornton model [43]. Our analysis used only RRs containing both the RR domain (the receiver domain) and the Myb DNA-binding domain. The ARR22 sequence (EMBL: At3g04280.1), a C-type RR from *Arabidopsis*, was used as outgroup [18]. The robustness of the tree was assessed by 1000 bootstrap replicates.

### Yeast two-hybrid interaction tests

The two-hybrid analysis was performed using a LexA DNA-binding domain encoding bait vector (pBTM116 referred as pLex) and a Gal4 activation domain encoding prey vector (pGADT7, Clontech). RR CDSs were cloned into the pGAD vector as *Xma*I*-sal*I (RR12, RR13, RR14, RR16, RR21, RR22), *Cla*I*-Xho*I (RR15) and *Cla*I*-Sal*I (RR19) fragments derived from PCR-amplified pGEMT-RR clones.

The yeast strain L40Δ (MATa ade2-101 his3-200 leu2-3,112 trp1-901 ura3-52 LYS2::(lexA op)x4-HIS3 URA3::(lexA op)x8-lacZ gal4Δ) was used for all transformations according to the lithium acetate method from Gietz *et al.*[53]. Co-transformed yeasts were selected onto leucine-trytophan lacking medium (−LW) for 4 days at 30°C then streaked onto leucine-trytophan-histidine lacking medium (−LWH) and grown for 4 days at 30°C. Due to autoactivation of HPts, 3-amino-1, 2, 4-triazole (3AT) was supplemented to -LWH medium at 60 mM (HPt2) or 20 mM (HPt7 and 9). X-Gal assays were performed according to the overlay method derived from Fromont-Racine *et al.*[54] by applying (directly onto the -LWH medium containing streaked positive yeasts) 10 mL of an X-Gal mixture containing agar (0.5%), phosphate buffer (0.25 M), SDS (0.1%) and X-Gal (0.04%). The blue colour was allowed to appear for 3h to 5h at 30°C. Interactions were tested using two different reporter genes (*HIS3* and *LacZ*) and all interactions were tested at least twice.

### ß-galactosidase activity measurements

Yeast colonies grown onto -LW medium were resuspended into Z buffer (Na_2_HPO_4,_ NaH_2_PO_4,_ KCl, MgSO_4_) at an OD_600_ between 0.5 and 0.7 and concentrated in a final volume of 300 μL. Cells were lysed by 3 cycles of liquid nitrogen/37°C incubations and 100 μL were used for triplicate tests. Two hundred μL of *ortho*-nitrophényl-ß-D-galactopyranoside (ONPG, 4 mg/mL) into Z buffer were added to the cells and the reaction was incubated at 37°C for 5 min, and then stopped with 200 μL of 1 M Na_2_CO_3_. One hundred μL of supernatant were transferred into a microplate for OD_420_ reading. Units of ß-galactosidase were expressed as Miller units. A minimum of three independent clones were used for each interaction HPt/RR tested. Data management and statistical analyzes were carried out with the statistical software SPPS 11.0 (SPSS, Chicago, IL, USA). For each variable, the normality of the distribution was tested by a Shapiro-Wilk test. Means are expressed with their standard error and compared by t-test or ANOVA followed by Scheffe test. All statistical tests were considered significant at P ≤ 0.05.

#### Interaction studies between HPts and B-type RRs by BiFC assays

BiFC assays were conducted using the pSPYNE(R)173 and pSPYCE(MR) plasmids [55] which allowed the expression of a protein fused to the C-terminus of the split-YFP fragments. For HPt2, HPt7, HPt9, RR16 and RR19, the cDNAs amplified using specific primers were cloned via *Spe*I in frame with the 3’ ends of the coding sequence of the N-terminal (YFP^N^, amino acids 1–173) and C-terminal (YFP^C^, amino acids 156–239) fragments of YFP. RR13 cDNAs were cloned via *Bam*HI in the same configuration. This led to the production of two distinct fusion proteins for HPt2, HPt7, HPt9, RR13, RR16 and RR19 which each type of fusion including YFP^N^-HPt2 and YFP^C^-HPt2 as described for HPt2. Transient transformation of *C. roseus* cells by particle bombardment and YFP imaging were performed using the CFP nucleus marker according to Guirimand *et al.*[56] with adaptation for BiFC assays [57].

### GFP-fused protein expression plasmids

To express the HPt-GFP and RR-GFP fusion proteins, the full coding sequence of HPts (HPt2 and 9) and RRs (RR13 and 16) were amplified by PCR using specific primers, extended by *Spe*I (HPt2 and 9, RR16) or *Xba*I (RR13) restriction sites at both extremities. The amplified cDNA was subsequently cloned into the *Spe*I *or Xba*I restriction site of pSCA-cassette GFPi [56] in frame with the 5’ extremity of the coding sequence of GFP.

Transient transformation of *C. roseus* cells by particle bombardment and YFP imaging were performed using the mcherry nuclear and nucleocytoplasmic markers according to Guirimand *et al*. [56].

### B-type RR transcripts analysis by RT-PCR

This study was done using the *Populus deltoides* (Bartr.) Marsh x *P. Nigra* L. clone Dorskamp genotype. The osmotic stress was imposed on one month old hydroponically grown rooted cuttings by supplementing the growth medium with PEG 6000 at 50 g/L [58]. Roots, stems, petioles and leaves were harvested and frozen after 0 and 10 minutes of stress. RNA extractions were carried out with the NucleoSpin® RNA Plant mini kit (Macherey-Nagel) according to the manufacturer’s instructions. One μg of total RNA was reverse transcribed using M-MuLV Reverse Transcriptase RNase H- (Finnzyme) according to the manufacturer’s procedure and used as template for PCR amplifications. All PCR reactions were performed in triplicate under optimal and comparable conditions. *Clathrin* was used as an expression control gene. The amplified fragments were separated by 1.2% agarose gel electrophoresis, stained with ethidium bromide and analyzed under UV light. Three independent biological experiments were performed.

## Competing interests

The authors declare that they have no competing interests.

## Authors' contribution

LB coordinated the project from its elaboration until the submission of the paper. She conducted technical experiments as the interaction study including two-hybrid assays, dosages of β-galactosidase activity and plasmid constructs for GFP/BiFC assays. She carried out the phylogenetic data analyzes, and wrote the paper. FC carried out the isolation of poplar B-type RRs and their expression profiles analysis. GG and VC were completely in charge of GFP/BiFC-imaging experiments. FB participated in the statistical analysis. FH co-supervised the LB PhD work. SC, supervisor of LB PhD, conceived of the study, carried out its coordination, performed part of the experiments and drafted the manuscript. FC, VC, CD, DM and FH helped in editing the manuscript, and all authors read and approved the final manuscript.

## Supplementary Material

Additional file 1: Table S1 Sequence of *Populus B-type RRs* and *Clathrin* genes specific primers used to achieve the co-expression analysis. Click here for file

Additional file 2: Table S2 Identifiers of B-type RRs proteins used in the phylogenetic analysis. Click here for file
